# The expression of CCAAT/enhancer binding protein (C/EBP) in the human ovary in vivo: specific increase in C/EBP**β** during epithelial tumour progression

**DOI:** 10.1038/sj.bjc.6690199

**Published:** 1999-03

**Authors:** K Sundfeldt, K Ivarsson, M Carlsson, S Enerbäck, P O Janson, M Brännström, L Hedin

**Affiliations:** Department of Physiology, Göteborg University, Göteborg, Sweden; Department of Obstetrics and Gynaecology, Göteborg University, Göteborg, Sweden; Department of Molecular Biology, Göteborg University, Göteborg, Sweden

**Keywords:** ovarian surface epithelial cells, tumour formation, transcription factors, C/EBP

## Abstract

The CCAAT/enhancer binding protein (C/EBP) family of transcription factors is involved in metabolism and differentiation of cells, especially in rodent liver cells and adipocytes. Their roles in vivo and in particular during pathophysiological conditions in humans are largely unknown. We have investigated the presence of C/EBPα, -β, -δ and -ζ in normal ovaries and in epithelial ovarian tumours of different stages. Immunohistochemical experiments demonstrated that C/EBPα and C/EBPβ were preferentially expressed in epithelial/tumour cells irrespective of stage or grade of the tumour. C/EBPβ was located in the nuclei of the cells, in contrast to C/EBPα, which was present only in the cytoplasm of these cells. The nuclear localization of C/EBPβ indicates an active role of this transcription factor in tumour cells, whereas the cytoplasmic distribution suggests a more passive function of C/EBPα. C/EBPδ and -ζ demonstrated a more diverse distribution with predominant localization to epithelial cells, but stromal distribution was also noted. The intracellular distribution was confined to both the nucleus and the cytoplasm for C/EBPδ and -ζ. Western blotting demonstrated that C/EBPα, -β, -δ and -ζ were present in a majority of the samples. The amount of C/EBPβ increased markedly with malignancy, i.e. with degree of dedifferentiation, while the other members of the C/EBP family displayed a more constant expression level. These results demonstrate an association between the expression of members of the C/EBP family and the formation of epithelial ovarian tumours, with C/EBPβ as a potential marker for these tumours. As C/EBPβ is known to be expressed during proliferation of cells in vitro, it may participate in the proliferative process of ovarian epithelial tumour cells in vivo and play a central role in tumour progression. © 1999 Cancer Research Campaign


*British Journal of Cancer* (1999) **79**(7/8), 1240–1248
© 1999 Cancer Research Campaign
Article no. bjoc.1998.0199

### The expression of CCAAT/enhancer binding protein (C/EBP) in the human ovary in vivo: specific increase in C/EBPβ during epithelial tumour progression

**K Sundfeldt[1], K Ivarsson[2], M Carlsson[1], S Enerbäck[3], PO Janson[2], M Brännström[2] and L Hedin[1]**

[1]Department of Physiology, [2]Department of Obstetrics and Gynaecology and [3]Department of Molecular Biology, Göteborg University, Göteborg, Sweden

**Summary** The CCAAT/enhancer binding protein (C/EBP) family of transcription factors is involved in metabolism and differentiation of cells, especially in rodent liver cells and adipocytes. Their roles in vivo and in particular during pathophysiological conditions in humans are largely unknown. We have investigated the presence of C/EBPα, -β, -δ and -ζ in normal ovaries and in epithelial ovarian tumours of different stages. Immunohistochemical experiments demonstrated that C/EBPα and C/EBPβ were preferentially expressed in epithelial/tumour cells irrespective of stage or grade of the tumour. C/EBPβ was located in the nuclei of the cells, in contrast to C/EBPα, which was present only in the cytoplasm of these cells. The nuclear localization of C/EBPβ indicates an active role of this transcription factor in tumour cells, whereas the cytoplasmic distribution suggests a more passive function of C/EBPα. C/EBPδ and -ζ demonstrated a more diverse distribution with predominant localization to epithelial cells, but stromal distribution was also noted. The intracellular distribution was confined to both the nucleus and the cytoplasm for C/EBPδ and -ζ. Western blotting demonstrated that C/EBPα, -β, -δ and -ζ were present in a majority of the samples. The amount of C/EBPβ increased markedly with malignancy, i.e. with degree of dedifferentiation, while the other members of the C/EBP family displayed a more constant expression level. These results demonstrate an association between the expression of members of the C/EBP family and the formation of epithelial ovarian tumours, with C/EBPβ as a potential marker for these tumours. As C/EBPβ is known to be expressed during proliferation of cells in vitro, it may participate in the proliferative process of ovarian epithelial tumour cells in vivo and play a central role in tumour progression.

**Keywords:** ovarian surface epithelial cells; tumour formation; transcription factors; C/EBP

The CCAAT/enhancer binding protein (C/EBP) family of transcription factors is a group of six known proteins: C/EBPα (C/EBP), C/EBPβ (NF-IL6, LAP), C/EBPγ (Ig-EBP), C/EBPδ (NF-IL6β), C/EBPε (CRP-1) and C/EBPζ (CHOP, GADD153) (Vinson et al, 1993). These proteins consist of three domains: a DNA-binding domain, a regulatory domain and a dimerization domain. The dimerization domains of the different family members have a high degree of homology. This domain is a leucine-zipper motif that allows the C/EBP to dimerize as both homo- and heterodimers. They can also form heterodimers with other leucine-zipper proteins in the Fos/Jun family and the ATF/CREB family (Vinson et al, 1993). The DNA-binding regions of the dimerized proteins recognize a palindromic CCAAT motif in the promoter of target genes. However, heterodimerization of any of the family members with C/EBPζ directs the complex away from this binding site because C/EBPζ has a different DNA-binding domain (Ron and Habener, 1992). There are also shorter forms of C/EBPα and -β, which lack this DNA-binding domain. These truncated forms may also act as inhibitors of transcriptional activation (Descombes and Schibler, 1991; Lin et al, 1993).

The roles of the C/EBP family in the control of proliferation and differentiation have mostly been studied in vitro in adipocytes (Cao et al, 1991; Umek et al, 1991). These in vitro studies demonstrated that the β- and δ-forms were active during the proliferative stages in the transition of preadipocytes into adipocytes prior to the activation of C/EBPα, which was expressed exclusively in the terminally differentiated cells. A constitutive expression of C/EBPα was also necessary to maintain adipocytes in their differentiated stage, when C/EBPα also acted as an antimitotic factor (McKnight, 1991; Samuelsson et al, 1991). C/EBPζ has also the potential to act as an inhibitor of the transcription by blocking DNA binding of other C/EBPs through dimerization (Ron and Habener, 1992). This dimerization results in a reduction of the differentiation process in vitro (Batchvarova et al, 1995).

The C/EBP family is also involved in the differentiation process of other cell types, e.g. intestinal epithelium (Chandrasekaran and Gordon, 1993), ovarian follicles (Piontkewitz et al, 1993), type II alveolar cells of the lung (Li et al, 1995) and myeloid cells (Scott et al, 1992). The involvement of these transcription factors in human diseases is largely unknown, although a fusion protein containing C/EBPζ has been reported in myxoid liposarcoma (Åman et al, 1992).

Ovarian tumours are the cause of 6% of deaths from malignancies in women in the Western hemisphere (Silverberg, 1984). Over 80% of these cancers originate from the surface (germinal) epithelium of the ovary (OSE cells). These carcinomas are suggested to originate from transformed OSE cells, either as a consequence of the repeated degradation and reconstitution of the ovarian surface after each ovulation or as the result of the formation of inclusion cysts with entrapped OSE cells (Hamilton, 1992).

*Received 17 November 1997*
*Revised 13 May 1998*
*Accepted 13 July 1998*

*Correspondence to:* L Hedin, Department of Physiology, Göteborg University, PO Box 432, SE-405 30 Göteborg, Sweden

**Table 1** List of tissues analysed for C/EBPα, -β, -δ and -ζ

| Sample no. | | Type | Stage | α | β | δ | ζ |
|---|---|---|---|---|---|---|---|
| N1 | N | Normal, fertile | | W | W | W | W |
| N2 | N | Normal, fertile | | W | W | W | W |
| N3 | N | Normal, fertile | | W | | W | W |
| N4 | N | Normal, post-menopausal | | W | W | W | W |
| N5 | N | Normal, post-menopausal | | I | I | I | I |
| N6 | N | Normal, post-menopausal | | I | I | I | I |
| N7 | N | Normal, post-menopausal | | I | I | I | I |
| Tb1 | B | Adenoma | | I | I | I | I |
| T8 | B | Adenofibroma | | W | W | W | W |
| T12 | B | Adenofibroma | | W,I | W,I | W,I | W,I |
| T17 | B | Adenoma | | W | W | | W |
| T36 | B | Adenofibroma | | I | I | I | I |
| T5 | BL | Borderline adenofibroma | IA | W | W | W | W |
| T16 | BL | Borderline adenoma | IA | W,I | W,I | W,I | W,I |
| T20 | BL | Borderline adenoma | IA | I | I | I | I |
| T32 | BL | Borderline muc. adenoma | IA | I | I | I | I |
| T22 | M | h diff. ser. adenocarcinoma | III | I | I | I | I |
| T25 | M | h diff. ser. adenocarcinoma | III | I | I | I | I |
| T34 | M | h diff. muc. adenocarcinoma | IA | I | I | I | I |
| T9 | M | m/p diff. ser. adenocarcinoma | III | W,I | W | W,I | W,I |
| T15a | M | m/p diff. ser. adenocarcinoma | III | W,I | I | I | W |
| T15b | M | Metastasis t. 15a | III | W | | W | W |
| T29 | M | m diff. ser. adenocarcinoma | III | I | I | I | I |
| T30 | M | m diff. ser. adenocarcinoma | III | I | I | I | I |
| T31 | M | m diff. ser. adenocarcinoma | III | I | I | I | I |
| T11 | M | m/p diff. ser. adenocarcinoma | II | W | W | W | W |
| T10a | M | m/p diff. ser. adenocarcinoma | III | W | W | | W |
| T10b | M | Metastasis t. 10a | III | W | W | | W |
| T14 | M | p diff. ser. adenocarcinoma | III | W | W | W | W |
| T26 | M | p diff. ser. adenocarcinoma | II | I | I | I | I |
| T27 | M | p diff. ser. adenocarcinoma | IC | I | I | I | I |
| T33 | M | p diff. ser. adenocarcinoma | III | I | I | I | I |
| T35 | M | p diff. ser. adenocarcinoma | III | I | I | I | I |
| T18 | M | Undiff. adenocarcinoma | III | W,I | W,I | W,I | W,I |
| T28 | M | undiff. adenocarcinoma | I | I | I | I | I |

Abbreviations: W, Western blotting performed; I, immunohistochemistry performed; N, normal; B, benign; BL, borderline; M, malignant; h, highly; m, moderately; p, poorly; diff, differentiated; ser, serous; muc, mucinous.

We have studied the expression of four of the members of the C/EBP family members (C/EBPα, -β, -δ, -ζ) in tissue specimens of ovarian epithelial tumours of different grades and stages in order to elucidate possible roles of these transcription factors in tumour formation in vivo. The specimens were analysed for cell-specific localization by immunohistochemistry and further quantified by Western blotting.

### MATERIALS AND METHODS

### Human tissues

Biopsies from normal ovaries and tumour tissues of ovarian origin were obtained from 35 patients undergoing laparotomy (approved by the Ethics Committee of the Medical Faculty, Göteborg University). The material is described in Table 1. Tissues were immediately washed in ice-cold 0.9% sodium chloride, snap-frozen in liquid nitrogen and stored at –70° C until analysis. All samples were examined by two independent and experienced pathologists for diagnosis.

### Primary antibodies

The following antibodies/antiserum were used: C/EBPα (rabbit polyclonal, cat. no. sc-61 and a rabbit antiserum provided by SL McKnight, Dallas, TX, USA), C/EBPβ (rabbit polyclonal, cat. no. sc-150), C/EBPδ (rabbit polyclonal, cat. no. sc-151), C/EBPζ (GADD153) (rabbit polyclonal, cat. no. sc-793) and proliferating cell nuclear antigen (PCNA, mouse monoclonal, cat. no. sc-56). All antibodies were from Santa Cruz Biotechnology (San Diego, CA, USA). The antiserum against C/EBPα was used for immunohistochemistry (Piontkewitz et al, 1993). All C/EBP antibodies were diluted 1:500 for Western blotting and 1:100 for immunohistochemistry. The dilution of the PCNA antibody was 1:500. Cytokeratin AE1/AE3 monoclonal antibody was used at a dilution of 1:40 (Cat. no. 1124161, Boehringer Mannheim, Germany).

### Immunohistochemistry

Fresh-frozen tissues were cryosectioned and fixed in cold acetone at –20°C for 10 min, then dried at room temperature. The slides were then hydrated with cold phosphate-buffered saline (PBS) and blocked with 5% non-fat milk (NFM) for 30 min, before the addition of the primary antibodies diluted in buffer solution [PBS with 1% bovine serum albumin (BSA), 0.2% Triton X-100, 0.1% sodium azide], and left overnight at room temperature. Bound antibodies were visualized by biotinylated secondary horse anti-rabbit (C/EBP) or anti-mouse (cytokeratin) antibodies (Vector, Burlingham, CA, USA) and streptavidin–fluorescein isothiocyanate (FITC, Amersham, Buckinghamshire, UK). Sections were mounted with Moviol/Dabco mounting medium (0.4% Moviol, Hoechst, Frankfurt am Main, Germany) in 30% glycerol with the addition of 2.5% Dabco (4-diazabicyclo (2.2.2) octane; Fluka, Buchs, Switzerland) (Piontkewitz et al, 1993). In the control sections, which showed only negligible signals, the first antibody was replaced by 5% NFM (data not shown). The sections were viewed and photographed with a Nikon microphot FX fluorescence microscope. All sections were stained with an antibody against cytokeratin to verify the epithelial origin of cells.

### Western blotting

Soluble tissues were prepared by homogenization in a PE buffer (10 mM potassium phosphate buffer, pH 6.8, and 1 mM EDTA) containing 10 mM 3-(3-cholamidopropyl)dimethyl-ammonio 1-propane sulphate (CHAPS), aprotinin (200 kallikrein inhibitory units per ml), leupeptin (1 mg $ml^{-1}$), pepstatin (1 mg $ml^{-1}$) and Pefablock® (1 mg $ml^{-1}$) (Boehringer Mannheim, Germany). The homogenate was then sonicated (twice, for 15 s each time) and centrifuged (10 000 ***g***, 10 min, 4°C). Supernatants were stored at –70°C until analysis. The protein concentrations were measured according to Lowry. The samples were diluted in sodium dodecyl sulphate (SDS) sample buffer and heated at 95°C for 5 min before loading on a SDS-polyacrylamide gel (12% Tris-glycine) (NOVEX, San Diego, CA, USA). Fifty micrograms of total protein was loaded into each lane. The proteins were transferred to a polyvinyldifluoride membrane (Amersham, Buckinghamshire, UK) using a blotting system (NOVEX). The membrane was then incubated with specific antibodies. Prestained standards (SeeBlue, NOVEX) were used as weight markers. Immunoreactive protein was visualized by chemiluminescence using alkaline phosphatase-conjugated secondary goat anti-rabbit antibodies (dilution: 1:20 000) or goat anti-mouse antibodies (dilution 1:10 000) (Santa Cruz Biotechnology), and CSPD or CDP Star (Tropix, Bedford, MA, USA) as substrate. The membrane was exposed to ECL film

© Cancer Research Campaign 1999

(Amersham, Buckinghamshire, UK) at room temperature. For semiquantitative measurement of proteins on these Western blots, a software package (Quantity One, PDI, NY, USA) in The Discovery Series Densitometric Systems (Pharmacia Biotech, Lund, Sweden) with Desk Top Plus Scanner, were used. The optical density (OD) × mm from each band was measured.

Statistical difference was evaluated by *t*-test (two sample) and a value of $P < 0.05$ was considered to be significant (Altman, 1991).

### RESULTS

### C/EBPα

The immunohistochemical analyses included 23 samples (Table 1). C/EBPα was detected mainly as a cytoplasmic staining in all of the tumours and also in the normal ovaries. Nuclear staining was only apparent in adipose cells adjacent to the tumour tissue (Figure 1C). The expression was restricted to epithelial cells in most of the samples (Figure 2A, D), and staining of the stroma cells was demonstrated in only two of the samples (T31 and T52). CEBPα was also expressed in the epithelial cell layers of inclusion cysts (N5 and T12). The intensity of the staining was rather constant in the different tumour samples and did not appear to be related to stage or grade of the tumour.

The Western blotting analyses of C/EBPα included 17 samples (Table 1). The expression of C/EBPα was constant in all samples, with a slightly lower content in the undifferentiated tumour (Figure 5B). In addition to the expected band with a molecular weight of 42 kDa, a band of 30 kDa was detected in all samples, except in the undifferentiated tumour. This band represents a shorter form of the protein, lacking a part of its activating region (Lin et al, 1993). A constant band of approximately 50 kDa was also noted in all samples. This signal [C/EBP reactive material (CRM, Cao et al, 1991)] was unrelated to normal or tumour tissues. This band was also present in other cell types and species (data not shown).

### C/EBPβ

In the normal ovary, the OSE cells at the ovarian surface expressed minute amounts of C/EBPβ, whereas epithelial cells of cleft formations and inclusion cysts were positively stained (Figure 3). An intense staining was demonstrated in all tumours and the expression of C/EBPβ was restricted to the epithelial cells, regardless of the tumour type. Stromal staining was demonstrated in only four samples (T29, T31, T32 and T52), and this signal was less intense than in the epithelial cells. C/EBPβ expression was more pronounced in the malignant samples, although it was detected at all stages of dedifferentiation and in all grades (Figure 4). Furthermore, the immunohistochemical analyses of 22 samples (Table 1) revealed a different intracellular distribution of C/EBPβ compared with that of C/EBPα. C/EBPβ was localized to the nucleus in the majority of tumour cells in the malignant samples (Figures 1D, 4), in contrast to C/EBPα, which demonstrated mainly a cytoplasmic staining. In the inclusion cysts (N5 and T12), both cytoplasmic and nuclear staining was observed for C/EBPβ (Figure 3B).

The presence of C/EBPβ was investigated by Western blotting in 14 samples (Table 1). The intact, full-length protein was detected as two bands, at 34 kDa and 38 kDa. The two normal ovaries expressed C/EBPβ, while the benign and borderline samples contained minute amounts of the protein. All the malignant samples contained large amounts of C/EBPβ (Figure 5A). In fact, the expression in the malignant samples was significantly higher ($P < 0.01$) than in normal ovaries and benign/borderline tumours. In addition, the malignant tumours contained a band at 18 kDa, representing the shorter form of the protein, LIP (Descombes and Scibler, 1991). The pattern of expression of C/EBPβ with an increased content in the malignant tumours correlated with that of PCNA (Figure 6).

### C/EBPδ

Immunohistochemical analyses of 23 tissue sections (Table 1) for C/EBPδ revealed staining of epithelial cells, while stromal staining was detected in 13 of these tumours (56%). In some of the sections, the stromal signals appeared as dots (Figure 2B, E). C/EBPδ was detected in the nucleus in all samples (Figures 1E and 2B, E). The intensity of the staining was constant, although some malignant tumours exhibited a stronger signal.

The contents of C/EBPδ were analysed in 13 samples with Western blotting (Table 1). The band of 30 kDa representing this protein was present in all samples. The levels were variable and no pattern related to degree of differentiation or grade could be detected. The malignant tumours expressed an additional band of 36 kDa (Figure 5B).

### C/EBPζ

Immunohistochemical analyses included 22 samples (Table 1). The staining was predominantly localized to the epithelial areas of the tumours, although seven of the samples (32%) also demonstrated C/EBPζ signals in the stromal tissue (Figures 1F and 2C, F). A faint staining was also demonstrated in the epithelial cells of inclusion cysts. The intracellular localization of C/EBPζ was predominantly perinuclear in both epithelial and stromal cells. These signals sometimes appeared as rings or dashes (data not shown).

Western blotting of 17 samples (Table 1) demonstrated C/EBPζ as a 29-kDa band. The expression was slightly higher in the malignant tumours than in normal ovaries and benign tumours, although the difference was not as pronounced as for C/EBPβ. In addition, a band of approximately 20 kDa was visible in the malignant tumours (Figure 5B).

### DISCUSSION

We have investigated the presence of four members of the C/EBP family in epithelial ovarian tumours and normal ovaries. In this study, we found that the expression of C/EBPβ was more pronounced in malignant tumours than in benign and borderline tumours. These differences in the contents were correlated with the expression of PCNA, a marker for cell proliferation (Takasaki et al, 1981), in the same samples. As C/EBPβ is known to be expressed in cells in vitro at proliferative and not fully differentiated stages, this finding also suggests a role for C/EBPβ during increased proliferation of tumour cells, similar to the proposed function of classical oncogenes and transcription factors in tumorigenesis.

The C/EBPβ protein was detected as two bands of the full-length protein (LAP) at 34 kDa and 38 kDa. Multiple forms of

 *© Cancer Research Campaign 1999*

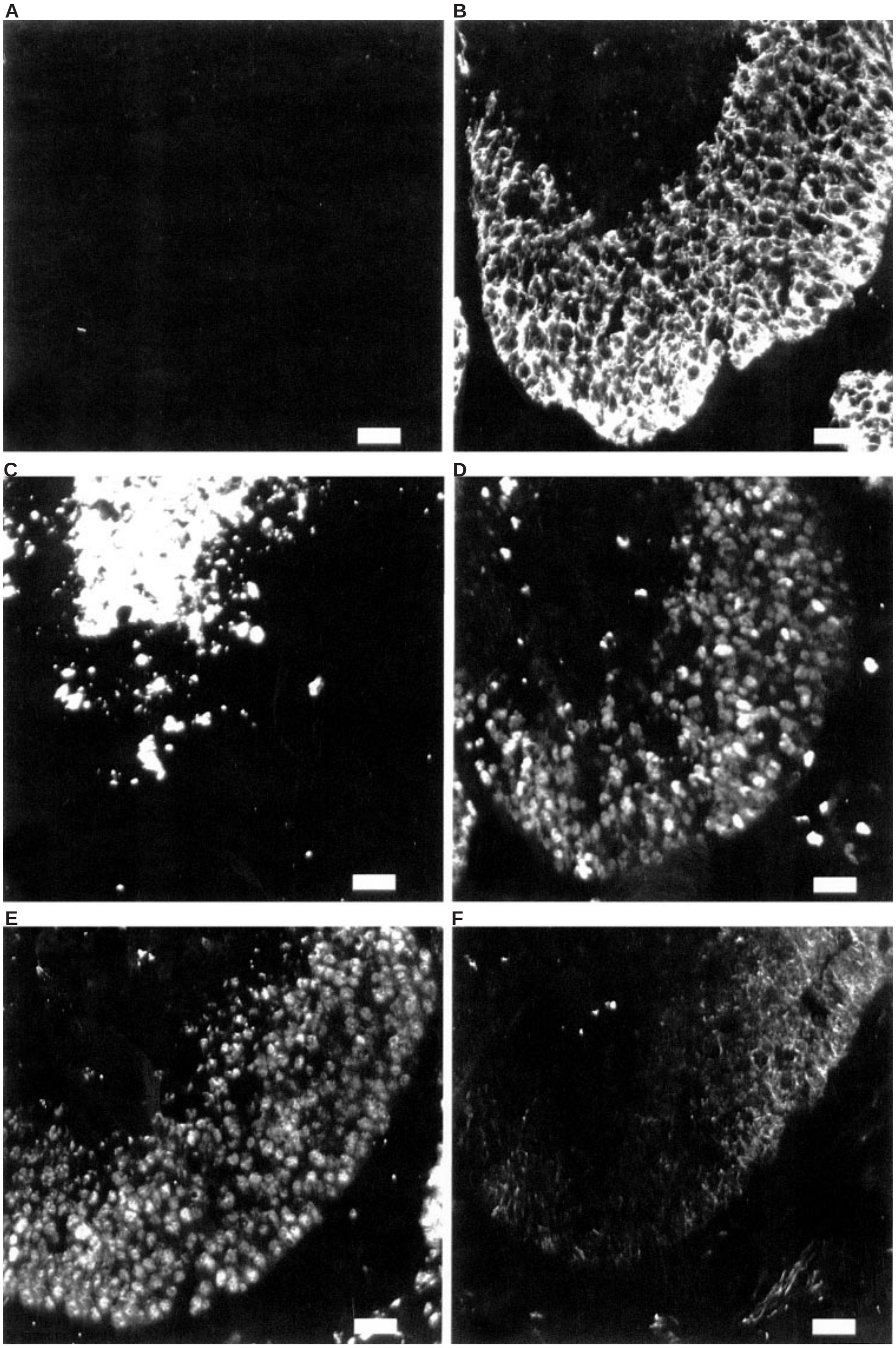

**Figure 1** Detection of four members of the C/EBP family with immunohistochemistry in a moderately differentiated serous adenocarcinoma (T31) (bar = 50 μm). (**A**) Negative control, (**B**) cytokeratin as a marker of epithelial cells, (**C**) C/EBPα, (**D**) C/EBPβ, (**E**) C/EBPδ and (**F**) C/EBPζ

LAP (30–40 kDa) were demonstrated in mouse mammary epithelial cells (Raught et al, 1995). The appearance of such forms might be attributed to post-translational modifications, e.g. phosphorylations. In fact, C/EBPβ was demonstrated to be phosphorylated by cyclic AMP-dependent protein kinase in vitro (Park et al, 1993) and $Ca^{2+}$ calmodulin-dependent protein kinases in vitro (Wegner et al, 1992). The shorter form of C/EBPβ (LIP, 18 kDa) was detected only in the malignant samples, although this does not exclude the presence of small amounts of the proteins in the normal ovary and in the benign tumours. The two isoforms, LAP and LIP, are explained by differential use of two AUGs within the same transcript (Descombes and Schibler, 1991). LIP is probably not functional as an activator of transcription, because the activational domain in the N-terminal part of the protein is missing. More

© Cancer Research Campaign 1999

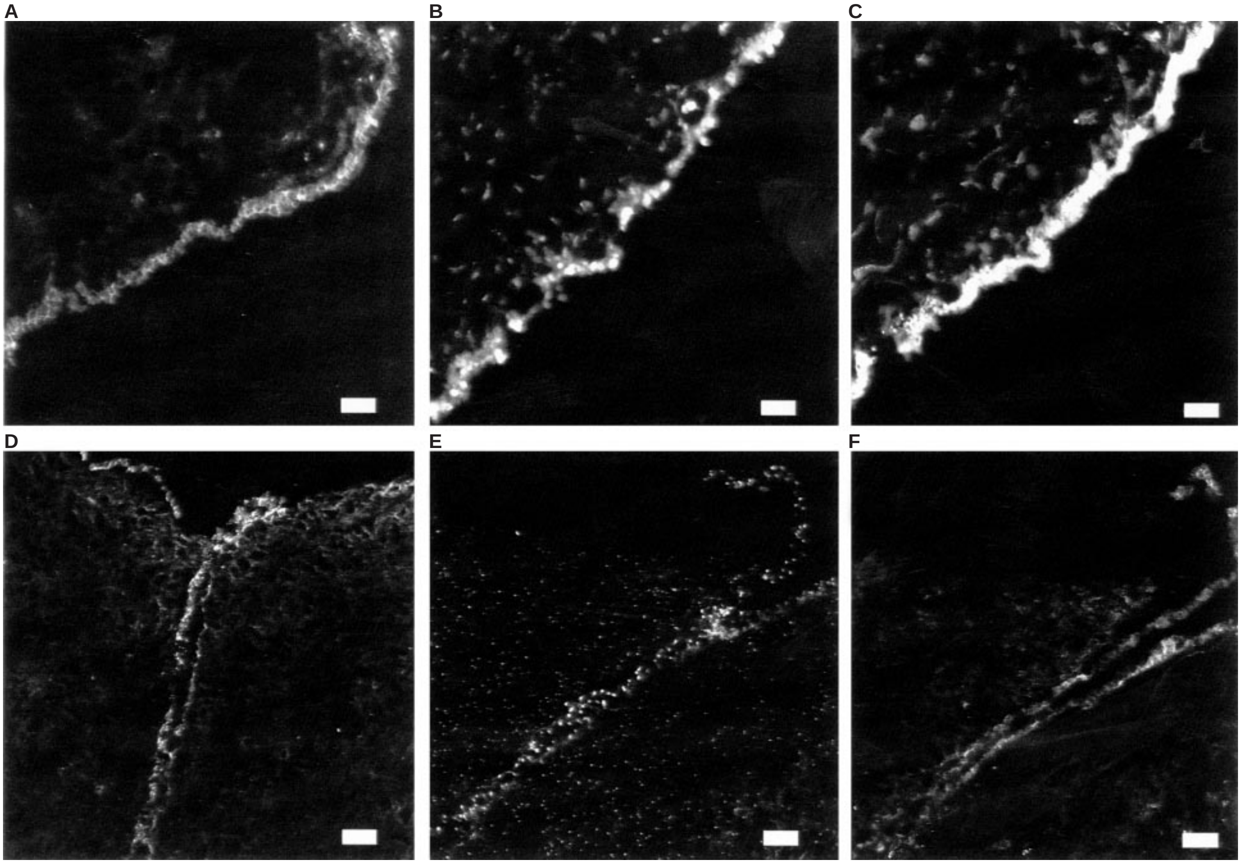

**Figure 2** Detection with immunohistochemistry (bar = 50 μm) of C/EBPα (**A** and **D**), C/EBPδ (**B** and **E**) and C/EBPζ (**C** and **F**), in a post-menopausal normal ovary (N5); surface epithelium (**A–C**), epithelial cells lining a cleft formation (**D–F**)

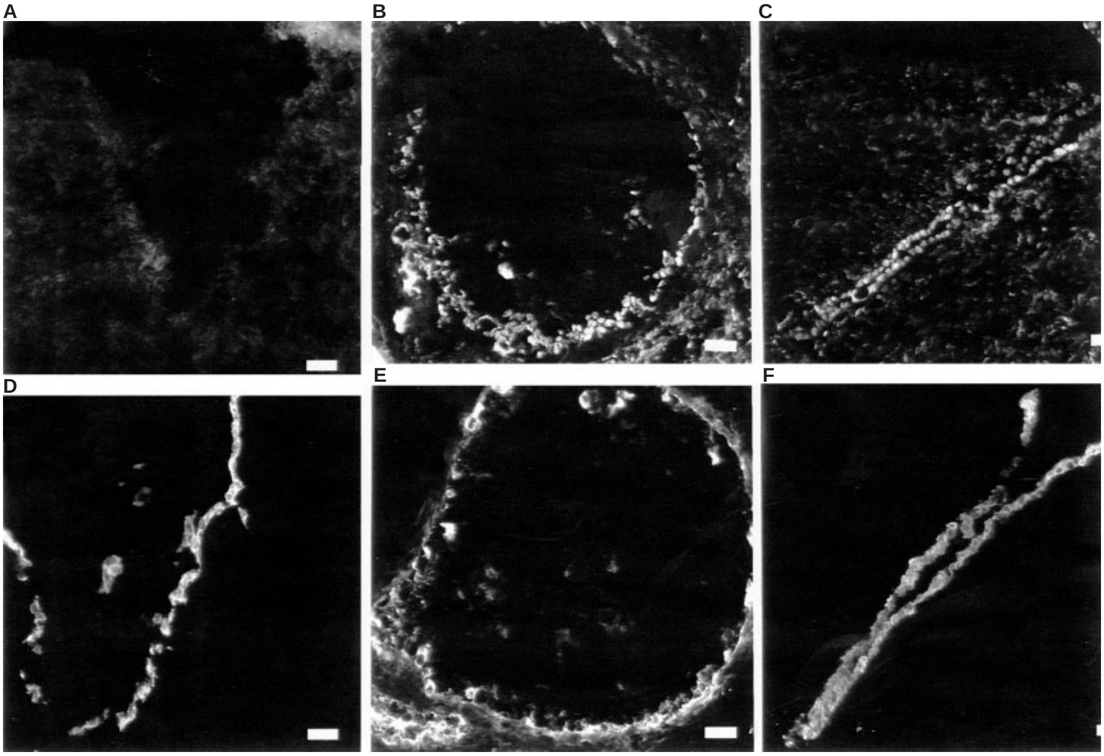

**Figure 3** Detection of C/EBPβ in a post-menopausal normal ovary (N5) with immunohistochemistry (bar = 50 μm) (**A**) surface epithelial cells, (**B**) inclusion cyst, (**C**) cleft formation and (**D–F**) corresponding tissue sections incubated with antibodies against cytokeratin, a marker of epithelial cells

*© Cancer Research Campaign 1999*

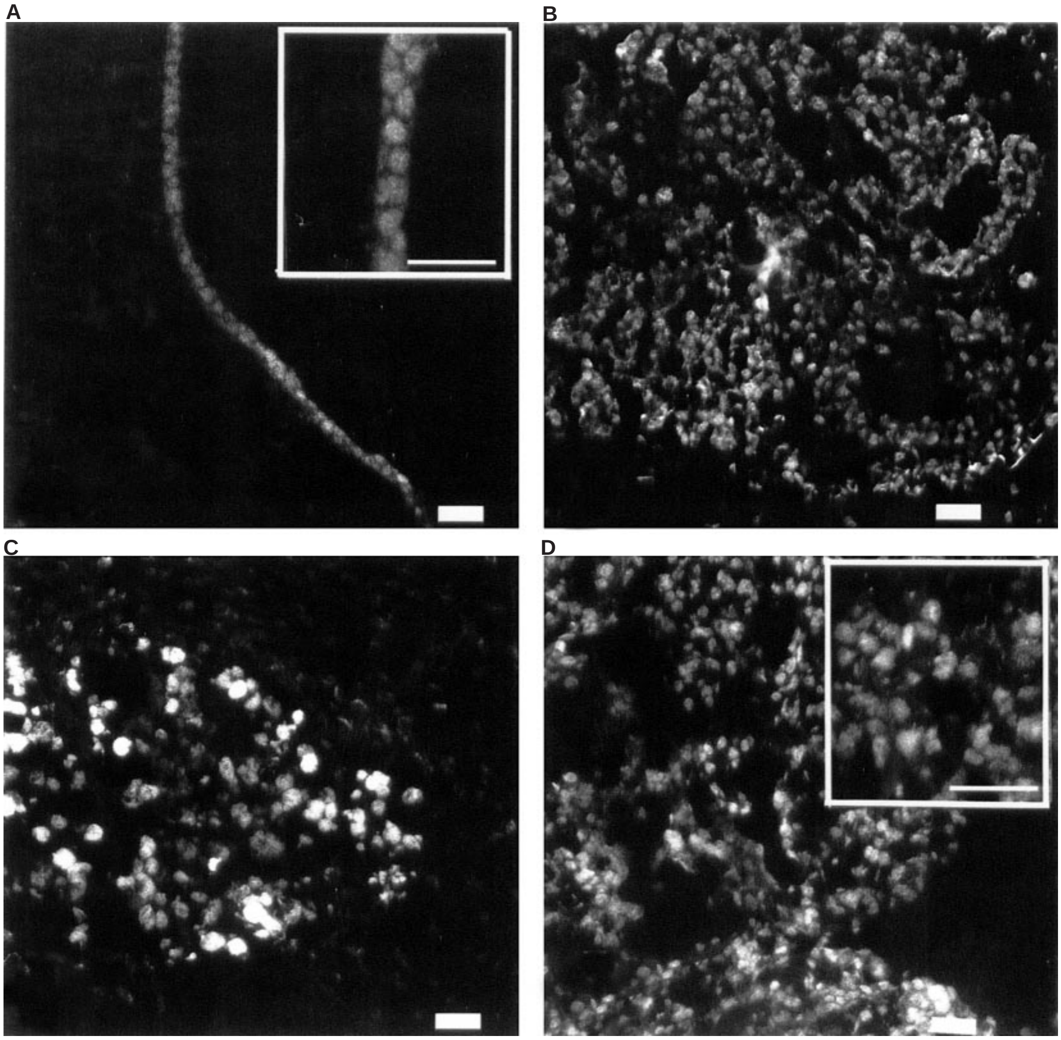

**Figure 4** Detection of C/EBPβ in (**A**) epithelial cells of a borderline tumour (T16), (**B**) moderately differentiated adenocarcinoma (T30), (**C**) poorly differentiated adenocarcinoma (T33) and (**D**) undifferentiated adenocarcinoma (T18) (bar = 50 μm)

likely, the shorter form represses the functions of C/EBPβ by the formation of inactive dimers with the full-length protein. The ratio between the two different forms of the C/EBPβ was suggested to be of importance for the transcriptional activity in mouse mammary epithelial cells (Raught et al, 1995).

The immunohistochemical analyses revealed that the increased expression of C/EBPβ was preferentially localized to epithelial cells within the tumours. However, invaginated OSE cells in cleft formations and epithelial cells lining cysts also stained positive for C/EBPβ. The stromal compartments of normal ovaries and of tumours stained negative almost without exception. The specific appearance of C/EBPβ in inclusion cyst is of interest for the pathogenesis of epithelial ovarian tumours since such cysts have been suggested to constitute the initial steps in tumour development (Hamilton, 1992). The present results suggest a putative role for C/EBPβ in the initial, proliferative process but also later in tumorigenesis of these cells in vivo. The low contents of C/EBPβ in benign tumours may indicate that these tumours represent a different trait in tumour formation without the potential of malignant transformation, or that their growth capacity is very low.

C/EBPβ was present in the nucleus of the epithelial cells in all samples. This was in contrast to C/EBPα which was mainly localized to the cytoplasm. The localization of C/EBPβ to the nucleus, together with the increased levels of this protein, proposes an active involvement in the transcriptional machinery of these cells. However, cytoplasmic localization of transcription factors, as noted for C/EBPα, may also reflect a regulatory pathway to inhibit transcriptional activity. For example, the activity of the transcription factor NFκB, is mainly regulated by shuttling of the protein between cytoplasm and nucleus (Ghosh and Baltimore, 1990). Such extranuclear localization of C/EBPα in malignant cells suggests an ‘inactive’ state of this transcription factor. In fact, translocation of C/EBPα, C/EBPβ and C/EBPδ between the cytoplasm and nucleus was regulated by tumour necrosis factor α (TNF-α) in hepatocytes in vitro (Yin et al, 1996). Interestingly, the expression of TNF-α in ovarian tumours was demonstrated to be positively correlated with tumour grade (Naylor et al, 1993). TNF-α has also been described as a growth factor for the ovarian tumour cells (Wu et al, 1993). These observations indicate that TNF-α could be involved in the post-translational regulation of C/EBP

*© Cancer Research Campaign 1999*

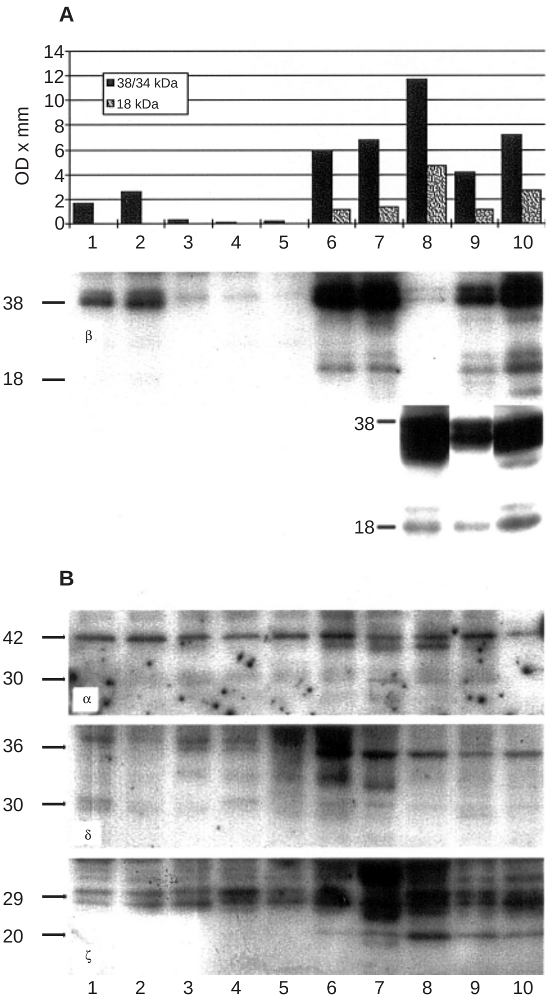

**Figure 5** Western blotting analysis of four members of the C/EBP family. (**A**) C/EBPβ (LAP 34 and 38 kDa, LIP 18 kDa) and the quantification by densitometry (OD × mm, the result of sample 8 refers to lower blot). (B) C/EBPα (42 kDa), C/EBPδ (30 kDa) and C/EBPζ (29 kDa). The samples: 1 and 2, normal post menopausal ovaries (N1, N4); 3 and 4, benign tumours (T12, T8); 5, a borderline tumour (T5); 6–9, medium to poorly differentiated malignant tumours (T11, T9, T10a, T14); and **10**, undifferentiated malignant tumour (T18)

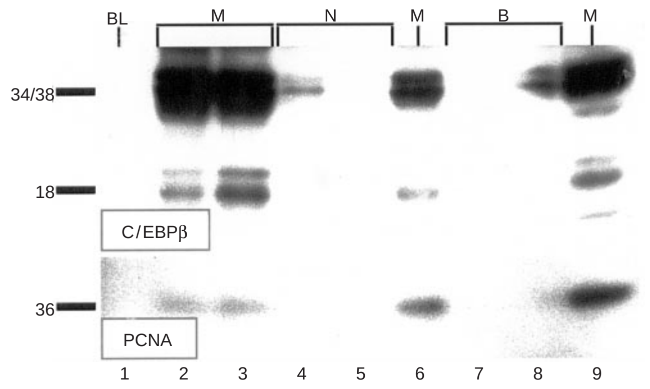

**Figure 6** Comparison of Western blots for C/EBPβ (LAP, 34/38 kDa and LIP, 18 kDa) (top) and PCNA (36 kDa) (bottom). The samples: 1, T5; 2, T10a; 3, T10b; 4, N1; 5, N2; 6, T14; 7, T16; 8, T17; 9, T18. Abbreviations: N, normal ovary; B, benign tumour; BL, borderline tumour; M, malignant tumour proteins in ovarian tumour cells. Studies have also revealed that the relocalization and transition of C/EBP into a nuclear, activated state was dependent on phosphorylation, mediated by cyclic AMP-dependent or $Ca^{2+}$-dependent protein kinases (Metz and Ziff, 1991).

C/EBPβ has mainly been found to induce expression of genes in acute-phase responses (Akira et al, 1990; Poli et al, 1990), such as granulocyte colony-stimulating factor (G-CSF) (Tanaka et al, 1995), interleukin (IL)-6 (Poli et al, 1990), and IL-8 (Matsusaka et al, 1993). Interestingly, IL-6 was expressed at high levels in ovarian tumours and stimulated proliferation of these cells (Watson et al, 1993). IL-8 was also measured in ovarian tumours, and the concentration of the protein was markedly higher in cyst fluid from malignant compared to benign tumours (Ivarsson et al, 1997). Both these cytokines, IL-6 and IL-8, were regulated by C/EBPβ in synergy with NFκB (Matsusaka et al, 1993). Furthermore, the expression of C/EBPβ could be stimulated by inflammatory substances, e.g. lipopolysacharides (LPS), IL-1 and IL-6 (Akira and Kishimoto, 1992). Recently, C/EBPβ was connected with proteins involved in the cell cycle, e.g. the tumour-suppressor retinoblastoma protein, Rb. Rb was demonstrated to directly interact and activate C/EBPβ (Chen et al, 1996). Loss or altered expression of Rb was suggested to be a primary event in many malignancies and this might also influence the activity of C/EBPβ. Studies in primary ovarian carcinomas demonstrated that Rb was normally expressed in a majority of these tumours (Dodson et al, 1994). Therefore, a direct interaction between Rb and C/EBPβ is plausible in epithelial ovarian tumour cells and suggests a role for C/EBPβ in the control of the cell cycle. In fact, a direct, cell cycle-regulated DNA-binding activity of C/EBPβ was demonstrated in regenerating rat hepatocytes in vivo (Rana et al, 1995).

Recently, the expression of the C/EBPβ isoforms (LAP and LIP) was examined in human breast tumours (Zahnow et al, 1997). It was found that LIP was present in malignant tumours, which stained negative for steroid receptors, suggesting that LIP might be useful as a prognostic marker for the identification of patients with a poor prognosis. C/EBPα was examined in liver carcinomas (Xu et al, 1994), where it was localized to both the nucleus and cytoplasm of the tumour cells. The expression of the protein was decreased when these tumours dedifferentiated. The anti-proliferating and anti-tumour properties of C/EBPα were demonstrated by induction of C/EBPα in human tumour cell lines (Timchenko et al, 1996; Watkins et al, 1996). This induction was mediated by increased levels of the cell cycle protein p21 (WAF-1), which is also regulated by the tumour suppressor p53.

In addition to the interaction with other factors for the transcriptional regulation of genes, the C/EBP proteins themselves affect the function of each other in a complex manner. The different members form both homo- and heterodimers, thereby extending and altering their regulatory potential. One of the proteins, C/EBPζ, has a different DNA-binding domain from the other members. Two amino acids in the basic region are replaced by prolines, which makes them unable to recognize the classic CCAAT sites. C/EBPζ can therefore act as a potent inhibitor of the other factors, directing them away from their target DNA (Ron and Habener, 1992). C/EBPζ was induced by cellular stress and inhibited proliferation with a subsequent arrest of growth in the $G_1$/S checkpoint. A fusion protein, TLS-CHOP, which inhibited the

*© Cancer Research Campaign 1999*

normal function of C/EBPζ (CHOP) was demonstrated in myxoid liposarcoma (Åman et al, 1992). The effect of C/EBPζ in differentiation was further investigated in 3T3-L1 cells that failed to convert into adipocytes. This effect was mediated by a reduction in C/EBPα gene expression (Batchvarova et al, 1995). In the present study, there was a minor increase in the concentration of C/EBPζ in the more malignant samples. As the immunohistochemical experiments revealed that most of the protein was localized outside the nucleus, this factor may exert some of its inhibitory potential in the cytoplasm, e.g. by binding to other leucine-zipper proteins and thereby blocking their entrance into the nucleus. An additional band of approximately 35 kDa was observed for C/EBPδ in the malignant tumours. Such a band might be attributable to extensive phosphorylation resulting in an altered function of the transcription factor (Park et al, 1993; Wegner et al, 1992). However, further studies are needed to reveal the pattern of phosphorylation of the different C/EBP proteins in tumour cells and to examine the effect of such modifications on their transcriptional activities.

In conclusion, the cell- and stage-specific pattern of expression of C/EBPs in epithelial ovarian tumours suggests an involvement of these transcription factors in tumour progression. Further studies are needed to determine their specific actions in the transcriptional machinery during dedifferentiation and proliferation of tumour cells. Thus, this family of transcription factors has the potential to be the target for both diagnostic and therapeutic development.

### ACKNOWLEDGEMENTS

This study was supported by the Swedish Medical Research Council (10375 and 12605 to LH), the Foundations of Magnus Bergvall, Ollie and Elof Ericsson, Assar Gabrielsson, Göteborg Medical Society, Hjalmar Svensson, Lundberg and King Gustav V Jubilee Clinic Cancer Research.

### REFERENCES

Akira S, Isshiki H, Sugita T, Tanabe O, Kinoshita S, Nishio Y, Nakajima T, Hirano T and Kishimoto T (1990) A nuclear factor for IL-6 expression (NF-IL6) is a member of a C/EBP family. *EMBO J* **9**: 1897–1906

Akira S and Kishimoto T (1992) IL-6 and NF-IL6 in acute-phase response and viral infection. *Immunol Rev* **127**: 25–50

Altman DG (1991) *Practical Statistics for Medical Research*. Chapman and Hall: London

Åman P, Ron D, Mandahl N, Fioretos T, Heim S, Arheden K, Willén H, Rydholm A and Mitelman F (1992) Rearrangement of the transcription factor gene CHOP in myxoid liposarcomas With t(12;16)(q13;p11). *Gene Chrom Cancer* **5**: 278–285

Batchvarova N, Wang X-Z and Ron D (1995) Inhibition of adipogenesis by the stress-induced protein CHOP (Gadd 153). *EMBO J* **14**: 4654–4661

Cao Z, Umek RM and McKnight SL (1991) Regulated expression of three C/EBP isoforms during adipose conversion of 3T3-L1 cells. *Genes Dev* **5**: 1538–1552

Chandrasekaran C and Gordon JI (1993) Cell lineage-specific and differentiation-dependent patterns of CCAAT/enhancer binding protein α expression in the gut epithelium of normal and transgenic mice. *Proc Natl Acad Sci USA* **90**: 8871–8875

Chen PL, Riley DJ, Chen-Kiang S and Lee WH (1996) Retinoblastoma protein directly interacts with and activates the transcription factor NF-IL6. *Proc Natl Acad Sci USA* **93**: 465–469

Descombes P and Schibler U (1991) A liver-enriched transcriptional activator protein, LAP, and a transcriptional inhibitory protein, LIP, are translated from the same mRNA. *Cell* **67**: 569–579

Dodson MK, Cliby WA, Xu HJ, DeLacey KA, Hu SX, Keeney GL, Li J, Podratz KC, Jenkins RB and Benedict WF (1994) Evidence of functional RB protein in epithelial ovarian carcinomas despite loss of heterozygosity at the RB locus. *Cancer Res* **54**: 610–603

Ghosh S and Baltimore D (1990) Activation in vitro of NF Kappa B by phosphorylation of its inhibitor I kappa B. *Nature* **344**: 678–682

Hamilton TC (1992) Ovarian Cancer, part 1: Biology. In *Curr Prob Cancer* **16**: 1–57

Ivarsson KU, Runesson EB, Haeger M, Sundfeldt K, Hedin LF, Janson PO and Brännström M (1999) The chemotactic cytokine interleukin-8 (IL-8) a cyst fluid marker for malignant human epithelial ovarian cancer. *Gynecol Oncol* (in press)

Li F, Rosenberg E, Smith CI, Notarfrancesco K, Reisher SR, Shuman H and Feinstein SI (1995) Correlation of expression of transcription factor C/EBP-α and surfactant protein genes in lung cells. *Am J Physiol* **269**: L241–L247

Lin F-T, MacDougald OA, Diehl AM and Lane MD (1993) A 30-kDa alternative translation product of the CCAAT/enhancer binding protein α message: transcriptional activator lacking antimitotic activity. *Proc Natl Acad Sci USA* **90**: 9606–9610

Matsusaka T, Fujikawa K, Nishio Y, Mukaida N, Matsashima K, Kishimoto T and Akira S (1993) Transcription factors NF-IL6 and NF-κB synergistically activate transcription of the inflammatory cytokines, interleukin 6 and interleukin 8. *Proc Natl Acad Sci USA* **90**: 10193–10197

McKnight SL (1991) C/EBP: A transcription factor that promotes terminal cell differentiation in a wide spectrum of tissues. In *Origins of Human Cancer: a Comprehensive Review*, Brugge J, Curran T, Harlow E and McCormick F (eds) pp. 345–352. Cold Spring Harbor Laboratory Press: Cold Spring Harbor

Metz E and Ziff E (1991) cAMP stimulates the C/EBP-related transcription factor rNFIL-6 to translocate to the nucleus and induce c-fos transcription. *Genes Dev* **5**: 1754–1766

Naylor MS, Stamp GW, Foulkes WD, Eccles D and Balkwill FR (1993) Tumour necrosis factor and its receptors in human ovarian cancer. Potential role in disease progression. *J Clin Invest* **91**: 2194–2206

Park EA, Gurney AL, Nizielski SE, Hakimi P, Cao Z, Moorman A and Hanson RW (1993) Relative roles of CCAAT/enhancer-binding protein beta and cAMP regulatory element-binding protein in controlling transcription of the gene for phosphoenolpyruvate carboxykinase (GTP). *J Biol Chem* **268**: 613–609

Piontkewitz Y, Enerbäck S and Hedin L (1993) Expression and hormonal regulation of the CCAAT enhancer binding protein-α during differentiation of rat ovarian follicles. *Endocrinology* **133**: 2327–2333

Poli V, Mancini FP and Cortese R (1990) IL-6DBP, a nuclear protein involved in interleukin-6 signal transduction, defines a new family of leucine zipper proteins related to C/EBP. *Cell* **63**: 643–653

Rana B, Xie Y, Mischoulon D, Bucher NL and Farmer SR (1995) The DNA binding activity of C/EBP transcription factor is regulated in the G1 phase of the hepatocyte cell cycle. *J Biol Chem* **270**: 18123–18132

Raught B, Liao WS-L and Rosen JM (1995) Developmentally and hormonally regulated CCAAT/enhancer-binding protein isoforms influence β-casein gene expression. *Mol Endocrinology* **9**: 1223–1232

Ron D and Habener JF (1992) CHOP, a novel developmentally regulated nuclear protein that dimerizes with transcription factors C/EBP and LAP and functions as a dominant-negative inhibitor of gene transcription. *Genes Dev* **6**: 439–453

Samuelsson L, Strömberg K, Vikman K, Bjursell G and Enerbäck S (1991) The CCAAT/enhancer binding protein and its role in adipocyte differentiation: evidence for direct involvement in terminal adipocyte development. *EMBO J* **10**: 3787–3793

Scott LM, Civin CI, Rorth P and Friedman AD (1992) A novel temporal expression pattern of three C/EBP family members in differentiating myelomonocytic cells. *Blood* **80**: 1725–1735

Silverberg E (1984) Cancer Statistics. *CA Cancer J Clin* **34**: 7–23

Takasaki Y, Deng JS and Tan EM (1981) A nuclear antigen associated with cell proliferation and blast transformation. *J Exp Med* **154**: 1899–1909

Tanaka T, Akira S, Yoshida K, Umemoto M, Yoneda Y, Shirafuji N, Fujiwara H, Suematsu S, Yoshida N and Kishimoto T (1995) Targeted disruption of the NF-IL6 gene discloses its essential role in bacteria killing and tumour cytotoxicity by macrophages. *Cell* **80**: 353–361

Timchenko NA, Wilde M, Nakanishi M, Smith JR and Darlington GJ (1996) CCAAT/enhancer-binding protein alpha (C/EBP-α) inhibits cell proliferation through the p21 (WAF-1/CIP-1/SDI-1) protein. *Genes Dev* **10**: 804–815

Umek RM, Friedman AD and McKnight SL (1991) CCAAT-enhancer binding protein: a component of differentiation switch. *Science* **251**: 288–292

Vinson CR, Hai T and Boyd SM (1993) Dimerization specificity of the leucine zipper-containing bZIP motif on DNA binding: prediction and rational design. *Genes Dev* **7**: 1047–1058

Watkins PJ, Condresay JP, Huber BE, Jacobs SJ and Adams DJ (1996) Impaired proliferation and tumorigenicity induced by CCAAT/ enhancer binding protein. *Cancer Res* **56**: 1063–1067

Watson JM, Berek JS and Martínez-Maza O (1993) Growth inhibition of ovarian cancer cells induced by antisense IL-6 oligonucleotides. *Gynecol Oncol* **49**: 8–15

© Cancer Research Campaign 1999 

Wegner M, Cao Z and Rosenfeld MG (1992) Calcium-regulated phosphorylation within the leucin zipper of C/EBP. *Science* **256**: 370–373

Wu S, Boyer CM, Whitaker RS, Berchuck A, Wiener JR, Weinberg JB and Bast RC (1993) Tumour necrosis factor alpha as an autocrine and paracrine growth factor for ovarian cancer: monokine induction of tumour cell proliferation and tumour necrosis factor alpha expression. *Cancer Res* **53**: 1939–1944

Xu L-X, Sui Y-F, Wang W-L, Liu Y-F and Gu J-R (1994) Immunohistochemical demonstration of CCAAT/Enhancer Binding Protein (C/EBP) in human liver tissues of various origin. *Chin Med J* **107**: 596–599

Yin M, Yang SQ, Lin HZ, Lane MD, Chatterjee S and Diehl AM (1996) Tumour Necrosis Factor α promotes nuclear localization of cytokine-inducible CCAAT/Enhancer Binding Protein isoform in hepatocytes. *J Biol Chem* **271**: 17974–17978

Zahnow CA, Younes P, Laucirica R and Rosen JM (1997) Overexpression of C/EBP–LIP, a naturally occurring, dominant–negative transcription factor, in human breast cancer. *JNCI* **89**: 1887–1891

*© Cancer Research Campaign 1999*
